# Ocular phenotypes associated with biallelic mutations in *BEST1* in Italian patients

**Published:** 2011-11-24

**Authors:** Andrea Sodi, Francesca Menchini, Maria Pia Manitto, Ilaria Passerini, Vittoria Murro, Francesca Torricelli, Ugo Menchini

**Affiliations:** 1 Department of Specialized Surgical Sciences, Eye Clinic, University of Florence, Italy; 2 Department of Ophthalmology, University of Udine, Italy; 3 Department of Ophthalmology, S.Raffaele Hospital, University of Milan, Italy; 4 Department of Genetic Diagnosis, Azienda Ospedaliero Universitaria Careggi, Florence, Italy

## Abstract

**Purpose:**

To report on the phenotype and the genotype of Italian patients carrying *BEST1* mutations on both alleles.

**Methods:**

Five Italian patients from four independent pedigrees with retinal dystrophy associated with biallelic *BEST1* variants were recruited from different parts of Italy. Molecular genetic analysis of the *BEST1* gene was performed with direct sequencing techniques. All the subjects included in the study were clinically evaluated with a standard ophthalmologic examination, fundus photography, optical coherence tomography scan, and electrophysiological investigations.

**Results:**

Six *BEST1* variants were identified. Three, c.1699del (p.Glu557AsnfsX52), c.625delAAC (p.Asn179del), and c.139C>T (p.Arg47Cys), were novel, and three had already been reported in the literature, c.301C>A(p.Pro101Thr), c.934G>A (p.Asp312Asn), and c.638A>G (p.Glu213Gly). Four were missense mutations, and two were deletions. Only one *BEST1* mutation was located within one of the four mutational clusters described in typical autosomal dominant Best vitelliform macular dystrophy (BVMD). Four patients showed a BVMD phenotype while one patient presented a clinical picture consistent with autosomal recessive bestrophinopathy (ARB).

**Conclusions:**

Biallelic *BEST1* sequence variants can be associated with at least two different phenotypes: BVMD and ARB. The phenotypic result of the molecular changes probably depends on the characteristics and the combination of the different *BEST1* mutations, but unknown modifying factors such as other genes or the environment may also play a role.

## Introduction

The human *BEST1* gene (OMIM 607854; previously known as *VMD2*) is located on the long arm of chromosome 11q12 and consists of 11 exons [1,2]. This gene is expressed predominantly in the retinal pigment epithelium (RPE) and encodes a 585-amino acid transmembrane protein, bestrophin-1, which localizes to the basolateral membrane of RPE cells [3]. The *BEST1* gene probably functions as a chloride channel but has also been considered an inhibitor of intracellular voltage-dependent Ca^2+^ channels [4,5].

Mutations in the *BEST1* gene have been reported in association with different ocular phenotypes [6]. The first disease shown to be caused by *BEST1* sequence variants was Best vitelliform macular dystrophy (BVMD) [2], a retinal disease characterized by a bilateral yellowish yolk-like lesion in the macula [7-9]. *BEST1* mutations may also be associated with several other eye diseases, including adult-onset vitelliform macular dystrophy (AOVMD) [10], autosomal dominant vitreo-retinochoroidopathy (ADVIRC) [11], retinitis pigmentosa [12], and microcornea, retinal dystrophy, cataract, and posterior staphyloma (MRCS) syndrome [13].

Usually the disorders associated with *BEST1* mutations are inherited as dominant traits. Thus, in typical pedigrees the affected patients carry a *BEST1* mutation on one allele, and *BEST1* mutations on both alleles are uncommon. Nevertheless, isolated patients with a biallelic mutation in *BEST1* and an apparent BVMD phenotype have occasionally been reported in previous studies on various ethnic groups [10,14-17]. A distinct retinal disorder, named autosomal recessive bestrophinopathy (ARB), has recently been described in association with biallelic *BEST1* mutations [18,19]. ARB is characterized by RPE dystrophy throughout the posterior pole, associated with retinal edema. A *BEST1* homozygous change was reported in four patients of the same family of Pakistani origin; their phenotype was interpreted as retinitis pigmentosa even though it showed some clinical similarities with the ARB phenotype [12]. Recently limited series of patients with different phenotypes and carrying biallelic *BEST1* sequence variants have been reported [20-22]. Therefore, the ocular phenotype of patients carrying a *BEST1* mutation on both alleles is still undefined. In the present paper, we report on the phenotype and the genotype of five Italian patients from four different pedigrees who carry *BEST1* mutations on both alleles.

## Methods

### Clinical evaluation

We first evaluated the medical records of all the patients whose blood samples were sent to the Laboratory of the Department of Genetic Diagnosis, Azienda Ospedaliera Careggi, Florence, Italy, for molecular genetic analysis of the *BEST1* gene. Patients harboring two *BEST1* mutations (one for each allele) were invited, together with their close relatives, for a detailed clinical examination and for a careful evaluation of their personal and family history.

**Figure 1:**
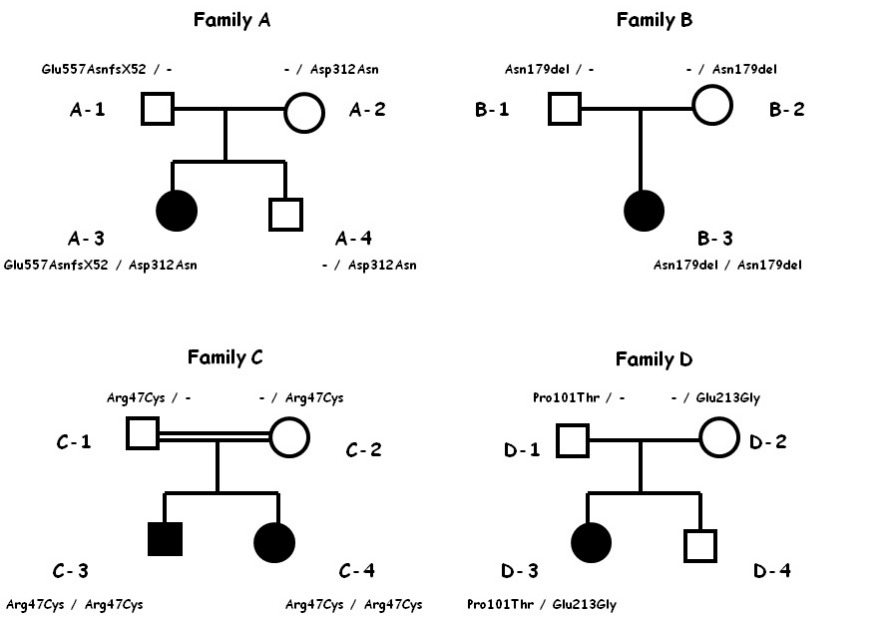
Pedigrees of the four families included in the study, with segregation of mutations. All affected patients carry biallelic *BEST1* mutations.

Five patients from four independent pedigrees were included in the study (Figure 1). Two were enrolled through the Hereditary Retinal Degenerations Referring Center of the Eye Clinic, University of Florence; one was regularly followed at the Eye Clinic of the University of Udine, and two patients were recruited through the Genetic Eye Disease Clinic of the Ospedale S.Raffaele, University of Milan, Italy. Interestingly, the families of both patients from the Florence unit originated from northeastern Italy.

The study adhered to the tenets of the Declaration of Helsinki and was approved by the local ethics committees. Moreover, each patient gave written informed consent for his or her involvement.

All the subjects included in the study were clinically evaluated with a standard ophthalmologic examination, fundus photography (Zeiss Retinograph Carl Zeiss, Dublin, CA, in Florence; TRC 50 IX camera and acquisition software Imagenet 2000, Topcon Optical Co., Tokyo, Japan, in Udine and Milan), optical coherence tomography (OCT) scan (Topcon 3D OCT-1000, Topcon Medical Systems Inc., Oakland, NJ, in Florence, Stratus Tomographer, model 3000; Carl Zeiss Meditec, Inc., Humphrey Division, Dublin, CA, in Udine, and Spectral OCT SLO OPKO/OTI, Miami, FL, in Milan), electroculogram (EOG), and electroretinogram (ERG). Electrophysiological investigations (Retimax, Roland Consult, Brandenburg, Germany, in Florence; Veris System, EDI, Inc., San Mateo, CA, in Udine; EREV 2000 LACE Elettronica, Pisa, Italy in Milan) were performed according to the existing ISCEV Guidelines [23,24]. Because of the young age of some of the patients, fluorescein angiography and fundus autofluorescence were performed only in selected cases.

### DNA extraction and PCR amplification

After informed consent and a complete family history were received, 10 ml of peripheral blood were obtained from the antecubital vein using EDTA-containing vials. DNA was extracted from 200 μl peripheral using an automated method involving the BioRobot EZ1 workstation (QIAGEN GmbH, Germany).

PCR amplification of 11 exons and flanking intronic regions of the *BEST1* gene was performed using 50–100 ng of genomic DNA. Amplification was performed in 50 mmol/l KCl, 10 mmol /l Tris-HCl, pH 8.3, 5 mmol/l MgCl_2_, 200 μmol/l dNTPs, and 0.5 μmol/l; for each primer set, AmpliTAq DNA polymerase (1 Unit of Ampli TAq Gold; Applied Biosystems, Foster City, KA) was added for each 25 μl reaction. PCR was performed with a multiblock MWG PCR System; cycling parameters for the reactions were optimized for each exon: 95 °C for 13 min and then 30 cycles of 95 °C for 1 min; “x” °C for 1 min; 72 °C for 51 min (for which x °C is the primers’ annealing temperature) with a final extension of 72 °C for 10 min. The primers used are shown in Table 1. PCR amplification was performed using the Core System-Robotic Station (Beckman Coulter). PCR products were purified with a Biomek NX station (Beckman Coulter).

**Table 1:** Primers used for the PCR amplification of the BEST1 gene.

**Exon**	**Sense primer (5′-3′)**	**Antisense primer (5′-3′)**	**Annealing temperature (°C)**
1	ACCCAACACCCTCCAAGAA	AGTCCACCTGGGGCACCTT	60
2	CCCTACAAACCCCCAATCG	CCAGCCACATCCTTCCCAG	60
3	AGTCTCAGCCATCTCCTCGC	TGGCCTGTCTGGAGCCTG	60
4	AGAAAGCTGGAGGAGCCGA	TTCACCCATCTTCCATTCCT	58
5	GCCATCCCTTCTGCAGGTT	GCGGCAGCCCTGTCTGTAC	62
6	GGGGCAGGTGGTGTTCAGA	GGCAGCCTCACCAGCCTAG	60
7	TGATTTCAGGGTTCCCACCTAG	CATCCTCGTCTCAGGCAGCT	65
8	AGGGTTTACAGAGCCTCACCTG	CACTTTGGGGAAGGTCCATG	60
9	CCTCCAAGTCATCAGGCACATA	CTAGGCAGACCCCTGCACTAG	65
40817	ACTGGCTCAGCCCTGCATC	GTGGGGCACTGTAGTAGCCTG	60
40818	CCTTCAAGTCTGCCCCACTG	TGCAGTGCCCCTGGTTC	60
11	GGTACCTTCCATACTTATGCTG	AGGTCTTGGGATGAATCAGA	60

For each exon the forward primer, the reverse primer, and the annealing temperature are indicated.

### Mutational analysis

Standard cycle-sequencing reactions with BigDye Terminator Mix v1.1 (Applied Biosystems) contained 3–10 ng of purified PCR products in 20 μl and were performed with forward and reverse primers for the initial amplification. The sequencing reactions were precipitated, dried, and then sequenced on a sequencer 3730 DNA Analyzer. Finally, data obtained from the Sequence Analysis Software (Applied Biosystems) were aligned with the wild-type *BEST1* gene sequence (GenBank Database). According to the EMQN Best Practice Guidelines, a sequence mismatch was considered a disease-causing mutation only if absent in 300 healthy controls, associated with amino acidic change, and confirmed with a new independent PCR.

Alamut 1.5 software (Interactive Biosoftware, Rouen, France) was used to interpret the unclassified variants.

## Results

### Family A

A 6-year-old child (patient A-3) with a history of macular dystrophy and reduced vision was referred to the Eye Clinic of the University of Florence in May 2006. On examination, the best corrected visual acuity (BCVA) was 20/32 right eye (OD) and 20/50 left eye (OS). Fundus examinations revealed a vitelliform disc in the vitelliruptive stage in the macular area in both eyes (OU). In OS, two extrafoveal additional vitelliform discs were detected in the temporal area at the posterior pole. EOG showed an Arden ratio of 1.00 OD and 1.12 OS. The photopic electroretinographic (ERG) response was within normal limits while scotopic ERG showed a reduced amplitude (about 25% compared with the average values of the control group). In January 2008, the young patient developed choroidal neovascularization (CNV) with macular hemorrhage in OS and received two photodynamic therapy (PDT) treatments.

**Figure 2:**
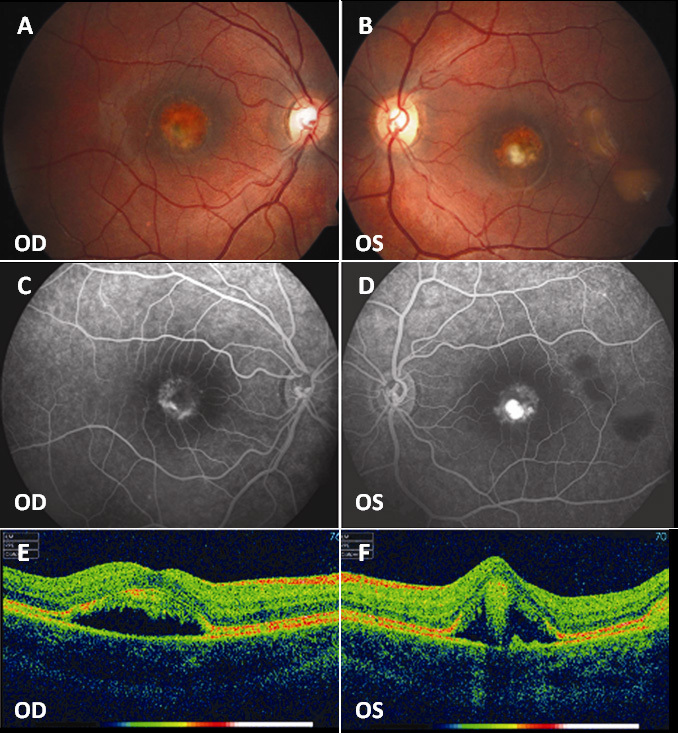
Fundus photographs, fluorescein angiography, and optical coherence tomography (OCT) scans of patient A-3. **A**, **B**: Bilateral vitelliform disc in the vitelliruptive stage resulting in (**C**, **D**) a macular hypofluorescent lesion with irregular areas of hyperfluorescence and (**B**) smaller multifocal vitelliform discs resulting in (**D**) hypofluorescent areas in left eye (OS). **E**, **F**: Bilateral macular optically empty lesions with clumping of hyperreflective material on the posterior retinal surface and some irregular thickening of the RPE layer. OD represents the right eye.

At the last examination in July 2010, BCVA was 20/50 OD and 20/100 OS. In OD, fluorescein angiography showed a round hypofluorescent area due to the vitelliform disc, with irregular areas of hyperfluorescence related to localized RPE atrophy, without dye leakage; in OS, hyperfluorescent abnormalities were detected in the macular area, in association with staining of the fibrotic areas, without any sign of dye leakage (Figure 2). OCT scans passing through the macula showed in OU an optically empty lesion with clumping of hyperreflective material on the posterior retinal surface and some irregular thickening of the RPE layer (Figure 2). The patient’s father (patient A-1, age 43 years), mother (patient A-2, age 43 years), and younger brother (patient A-4, age 10 years) showed normal visual acuity, normal fundus appearance, and normal ERG. The EOG response was normal for the proband’s parents (the Arden ratio was 2.40 OD and 3.61 OS for the father, and 1.88 OD and 2.31 OS for the mother), while the brother showed a reduced Arden ratio (1.23 OD and 1.11 OS).

In the proband, molecular analysis detected two sequence variants of the *BEST1* gene in the heterozygous state: c.934G>A (p.Asp312Asn) already described in the literature in association with BVMD and the novel variant c.1699del (p.Glu557AsnfsX52) [10]. Both mutations were absent in 300 healthy controls of Italian origin. The second variant is a single base pair deletion and gives rise to a premature stop after 52 nucleotides; therefore, a pathogenic effect is likely. The variant could not be evaluated with the Alamut software, which provides a predictive evaluation only for missense mutations. The patient’s father carried the mutation c.1699del (p.Glu557AsnfsX52) in the heterozygous state and the mother and the brother the mutation c.934G>A (p.Asp312Asn) in the heterozygous state. The pedigree of the family and the segregation of the mutations are shown in Figure 1.

### Family B

**Figure 3:**
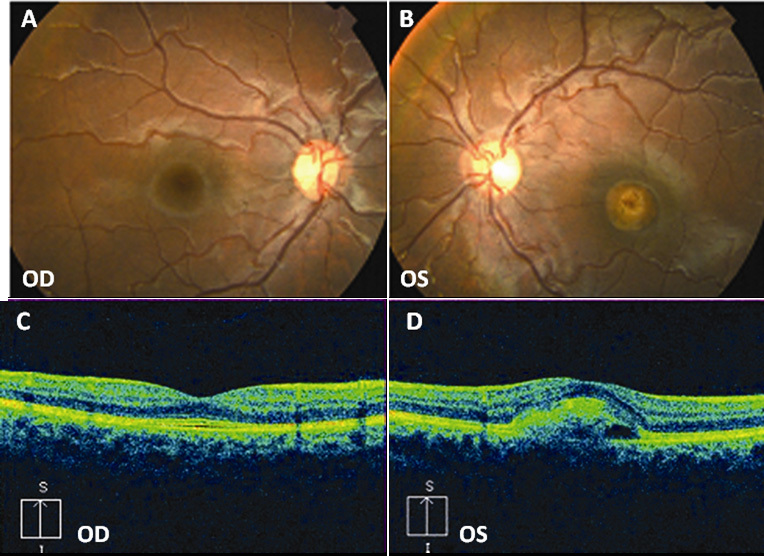
Fundus photographs and optical coherence tomography (OCT) scans of patient B-3. **A**: Normal fundus in right eye (OD) and (**B**) vitelliform disc in the vitelliruptive stage in left eye (OS). **C**: Normal macular profile in OD and (**D**) central RPE detachment with hyperreflective material in OS.

In 2006 patient B-3 was referred to the Eye Clinic of the University of Udine at the age of 6 because she complained of mild visual impairment. BCVA was 20/20 OD and 20/25 OS. Fundus appearance was normal in OD while OS showed a vitelliform disc at the posterior pole (Figure 3). EOG was abnormal in OU, with an Arden ratio of 1.07 OD and 0.89 OS. The amplitude of the ERG response was slightly reduced for all the components (about 30% compared with the average values of the control group). OCT scans showed a normal macular profile in OD and an accumulation of hyperreflective material between the RPE and the neuroretina in OS (Figure 3). The clinical picture remained stable until the last examination in May 2010.

The patient’s father (B-1, age 38 years) and mother (B-2, age 37 years) had normal visual acuity and normal fundus appearance, and their ERG and EOG responses were within normal limits (1.75 OD and 1.80 OS for the father and 1.78 OU for the mother). Molecular analysis detected in this patient a novel *BEST1* gene sequence variant, c.625delAAC (p.Asn179del), in the homozygous state. This mutation consists of an in-frame deletion of three base pairs and does not give rise to a premature stop. The mutation was absent in 300 healthy controls of Italian origin. The same mutation was detected in both parents in the heterozygous state (Figure 1). The mutation could not be processed by the Alamut software because its scoring systems do not provide a predictive evaluation for deletions.

### Family C

**Figure 4:**
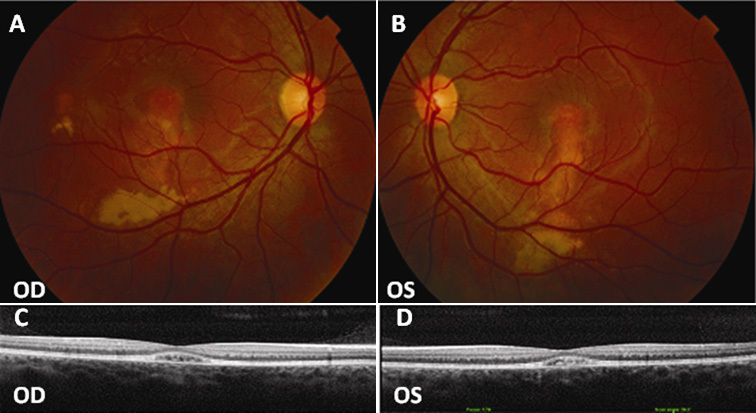
Fundus photographs and optical coherence tomography (OCT) scans of patient C-3. **A**, **B**: Bilateral vitelliform discs in the vitelliruptive stage with retinal pigment epithelium (RPE) dystrophy inferiorly to the macula. **C**, **D**: Macular optically empty lesions containing partially reabsorbed hyperreflective material. OD represents the right eye; OS represents the left eye.

The proband (patient C-3) was referred to the Eye Clinic of the University of Milan in 2004 at age 15 with a history of reduced vision in both eyes. At examination, BCVA was 20/40 OU. Fundoscopy showed at the posterior pole a vitelliform disc in the vitelliruptive stage with contiguous RPE dystrophy below the macula in OU (Figure 4). An OCT scan showed a bilateral macular optically empty zone containing partially reabsorbed hyperreflective material (Figure 4). ERG was within normal limits while EOG was abnormal with an Arden test of 1.20 OD and 1.54 OS. The clinical picture remained unchanged at the following ophthalmologic examinations in 2008 and 2010. The patient was the product of a consanguineous marriage. The parents underwent an ophthalmological examination in 2008, when the father (C-1) was 50 years old and the mother (C-2) was 47 years old; they had normal visual acuity and normal fundus appearance but were not available for electrophysiological testing. Molecular analysis detected in this patient a novel BEST1 gene sequence variant, c.139C>T (p.Arg47Cys), in the homozygous state. The same mutation was detected in both parents in the heterozygous state. The mutation was absent in 300 healthy controls of Italian origin. This novel mutation was located in a weakly conserved region of the protein, even though it was associated with a substitution involving two aminoacids with different chemical properties; the Alamut software classified the mutation as a tolerated change that may possibly affect the protein function, with a potentially low pathogenic effect.

**Figure 5:**
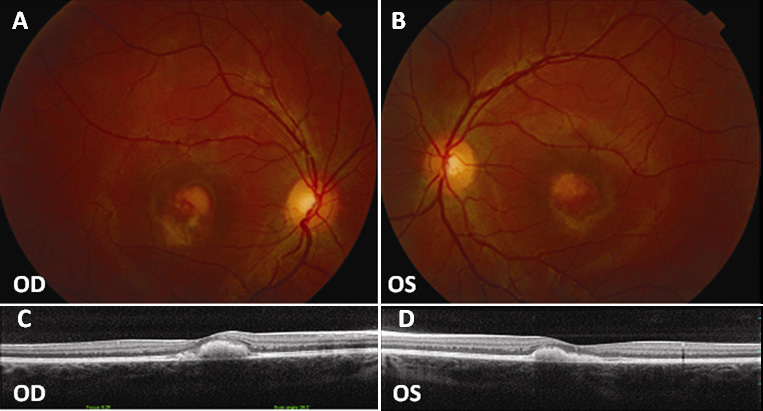
Fundus photographs and optical coherence tomography (OCT) scans of patient C-4. **A**, **B**: Bilateral vitelliform discs in the vitelliruptive stage. **C**, **D**: Bilateral hyperreflective dome-shaped lesions located between the RPE and the neuroretina. OD represents the right eye; OS represents the left eye.

The fourth patient (C-4) was the younger sister of the previous patient. She complained of bilateral visual reduction in 2008 at age 13. At examination, BCVA was 20/32 in both eyes. Fundus examination showed a bilateral vitelliform disc in the macular area (Figure 5). ERG was within normal limits, while the EOG showed an Arden test value of 1.65 OU. OCT scans showed a bilateral hyperreflective dome-shaped lesion located between the RPE and the neuroretina (Figure 5). The clinical picture was stable until the last examination in April 2010. The patient carried in the homozygous state the same *BEST1* sequence variant c.139C>T (p.Arg47Cys) found in the parents in the heterozygous state and in her elder brother in the homozygous state (Figure 1).

### Family D

**Figure 6:**
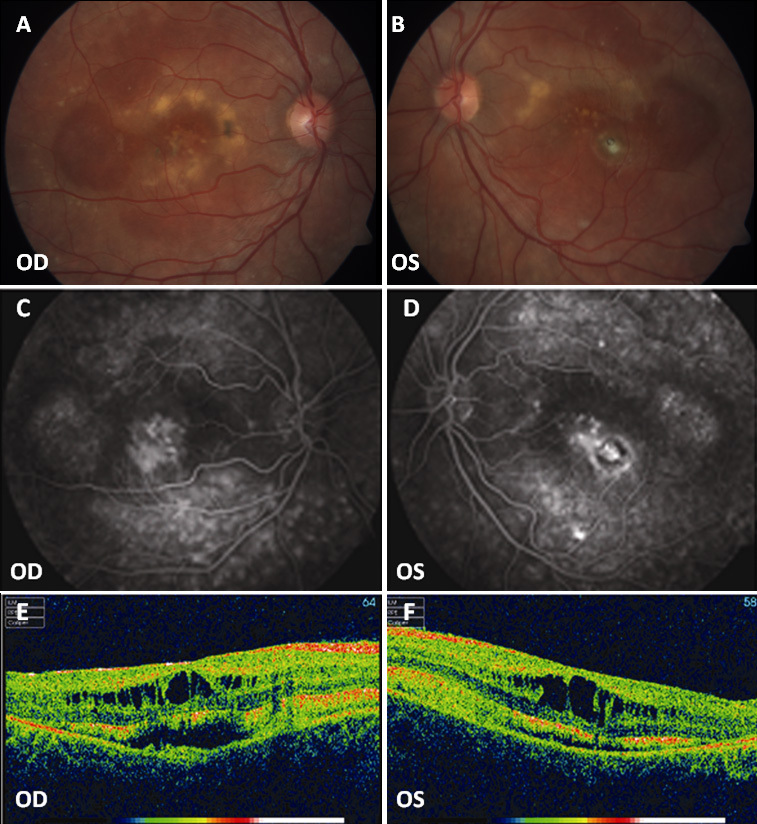
Fundus photographs, fluorescein angiography, and optical coherence tomography (OCT) scans of patient D-3. **A**, **B**: Widespread RPE dystrophy and yellowish deposits at the posterior pole with (**C**, **D**) bilateral macular hyperfluorescence and small hyperfluorescent spots at the posterior pole and mid-periphery. **E**, **F**: Bilateral macular cystoid spaces and two cleavage plans: the outer one between the retinal pigment epithelium (RPE) and the neuroretina and the inner one below the nerve fibers layer. OD represents the right eye; OS represents the left eye.

In January 2010, a 16-year-old girl (patient D-3) from northern Italy was referred to the Eye Clinic of the University of Florence with a history of reduced vision in both eyes that started when she was about 6 years old. At examination, her BCVA was 20/25 OD and 20/50 OS. Fundoscopy showed in OU diffused RPE dystrophy with yellowish deposits at the posterior pole and macular edema (Figure 6). The medical records of the patient demonstrated that no vitelliform disc had ever been detected during the course of the disease. An OCT scan showed bilateral macular cystoid spaces (Figure 6). Scotopic ERG was abnormal (amplitude was reduced by about 50% of the average value of the control group) while photopic ERG amplitude was slightly reduced (about 25% of the average values of the controls). The EOG Arden test values were 1.20 OD and 1.50 OS. Fluorescein angiography showed macular hyperfluorescence and small hyperfluorescent spots at mid-periphery, with a higher concentration around the optic disc (Figure 6). In 2008, the patient had received PDT for suspected CNV in OD.

The patient’s father (D-1, age 48 years) and mother (D-2, age 47 years) showed normal visual acuity, normal fundus appearance, and ERG response within normal limits. The mother’s EOG response was normal (the Arden ratios were 1.83 OD and 1.72 OS) while the father presented an Arden ratio of 1.35 OU. The patient’s younger brother (D-4) was examined in another hospital in 2004 when he was 5 years old; at that time, visual acuity and fundus appearance were normal. He was not available for further investigations.

In the proband, molecular analysis detected two sequence variants of the *BEST1* gene in the heterozygous state: c.301C>A (p.Pro101Thr) and c.638A>G (p.Glu213Gly); both have already been described in the literature in association with BVMD [25,26]. They were absent in 300 healthy controls of Italian origin. The patient’s father carried the mutation c.301C>A (p.Pro101Thr) in the heterozygous state and the mother the mutation c.638A>G(p.Glu213Gly) in the heterozygous state (Figure 1).

## Discussion

*BEST1* mutations are usually associated with dominant retinal disorders, and therefore homozygous or compound heterozygous mutations are rare [6]. We report on a series of Italian patients carrying mutations of the *BEST1* gene on both alleles.

Two of our patients were compound heterozygotes while three patients (two from the same family) were homozygotes. A significant clinical difference between the compound heterozygous and homozygous patients could not be detected in our series; compound heterozygous mutations were associated with a case of unilateral BVMD and with an ARB phenotype.

We identified six *BEST1* variants. Three were novel, and three have already been reported in the literature in association with BVMD, c.301C>A (p.Pro101Thr) [25] and c.638A>G (p.Glu213Gly) [26], or AOVMD, c.934G>A (p.Asp312Asn) [10]. Four were missense mutations, and two were deletions. Only one mutation, c.301C>A (p.Pro101Thr), was located within one of the four mutational clusters described in typical autosomal dominant BVMD [6]. Two of the detected mutations, c.934G>A (p.Asp312Asn) and c.139C>T (p.Arg47Cys), have already been reported in patients carrying another BEST1 mutation on the other allele [18,22]. Interestingly, the patient with c.934G>A (p.Asp312Asn) showed an ARB phenotype [18] while the c.139C>T (p.Arg47Cys) mutation (in the homozygous state) was associated with bilateral vertical elliptic vitelliform lesions at the posterior pole, a clinical picture more similar to classical BVMD [22]. Moreover, in our series two of the six mutations are located between aminoacids Arg 141 and Lys 289 of the bestrophin protein where a higher frequency of *BEST1* biallelic mutations have recently been reported [22].

The relatively high rate of novel mutations, the report of frequent deletions (rarely associated with the BVMD phenotype), the predominant location outside the BVMD mutational clusters, and the recurrence of some variants in the recessive form of the disease may suggest that certain mutations have a higher predisposition to be pathogenic when present on both alleles.

In our series, the biallelic BEST1 mutations are associated with two different phenotypes, BVMD and ARB. This agrees with recent reports of the association of homozygous or compound heterozygous BEST1 variants with different clinical pictures, ranging from the classical form of BVMD [20,22] to ARB [18,19,22], including some atypical phenotypes [21,22].

The clinical picture of patients A-3, B-3, C-3, and C-4 was consistent with BVMD, although with mild phenotypic differences. The patients had abnormal EOG while ERG was slightly abnormal in patients A-3 and B-3 and normal in the remaining patients. The relatives of patients A-3, B-3, C-3, and C-4 presented normal visual acuity, normal fundus appearance, and ERG response within normal limits. EOG was within normal limits for the parents of the patients A-3 and B-3 but showed a reduced Arden ratio for patient A-4, the younger brother of the proband A-3.

Isolated cases of patients with biallelic *BEST1* mutations and a BVMD phenotype have already been reported in literature [10,14-17].

In most of the cases, clinical and histopathological abnormalities were comparable to those found in heterozygous patients [15]. Similarly, our BVMD patients did not show a significantly more severe picture than that usually described in BVMD patients carrying a single *BEST1* mutation; nevertheless, mild ERG abnormalities were found in patients A-3 and B-3. The clinical expressivity was variable as the *BEST1* biallelic variants were associated with typical but also multifocal or unilateral BVMD phenotypes.

The heterozygous relatives of our BVMD patients did not have fundus or ERG abnormalities and showed normal EOG responses in most of the cases. Clinical data on heterozygous relatives of patients carrying *BEST1* biallelic mutations have not been reported in detail in the literature; we can only speculate from the available data that some heterozygous relatives may be clinically affected while others may show a normal fundus appearance [10,15-17]. In a recent paper, the heterozygous relatives of a patient with a homozygous *BEST1* mutation associated with a classical BVMD phenotype showed neither fundus nor EOG abnormalities [20].

The phenotype of patient D-3 was significantly different from those of the other patients, and it is consistent with the clinical diagnosis of autosomal recessive bestrophinopathy (ARB) [18,19].

This patient carried two *BEST1* mutations already described in the literature in association with BVMD. *BEST1* variants detected in association with the ARB phenotype have already been reported in BVMD [10,14] and in AOVMD [22]. These data are consistent with the hypothesis that in patients carrying biallelic *BEST1* mutations the resulting phenotype is determined by the interaction of the pathogenic effects of the different mutations on both alleles.

The abnormal EOG response of the patient’s father is not in agreement with previous reports where all the heterozygous carriers showed a normal EOG [18]. EOG abnormalities reported in isolated heterozygous cases of clinically healthy relatives of patients carrying biallelic *BEST1* mutations may be related to the influence of unknown genetic or environmental factors, even if technical problems or poor collaboration from the patient cannot be excluded.

Previous findings and our observations show that sequence variants of the *BEST1* gene on both alleles can be associated with at least two different phenotypes, BVMD and ARB. The phenotypic result of the molecular changes probably depends on the characteristics and the combination of the different *BEST1* sequence variants, but unknown modifying factors such as other genes or the environment may also play a role.

Because of this phenotypic heterogeneity, *BEST1* mutations may be involved in retinal diseases other than those already identified. A clearer understanding of the *BEST1* mutations spectrum and its associated phenotypes is warranted for better clinical evaluation, genetic counseling, and patient selection for possible future treatments.
